# Identification of novel tumor protein 63 variant associated with split-hand/foot malformation and tooth agenesis

**DOI:** 10.3389/fmed.2026.1811133

**Published:** 2026-04-24

**Authors:** Jie-Yi Long, Qin-Zhi Liu, Si-Hua Chang, Run-Yan Wang, Jie-Yuan Jin, Ning Li, Zhen-Bo Cheng

**Affiliations:** 1 Department of Hand and Microsurgery, Xiangya Hospital, School of Life Sciences, Central South University, Changsha, China; 2 School of Medicine, Shaoxing University, Shaoxing, China; 3 Physical Education Institute, Hunan University of Financial and Economic, Changsha, China; 4 Department of Clinical Laboratory, Hunan Provincial People's Hospital, The First Affiliated Hospital of Hunan Normal University, Hunan Normal University, Changsha, Hunan, China

**Keywords:** congenital limb deformity, SHFM, tooth agenesis, TP63, whole exome sequencing

## Abstract

**Background:**

Split-hand/foot malformation (SHFM) is a serious congenital anomaly. A multitude of pathogenic genes associated with SHFM have been identified; *TP63, DLX5, FGFR1*, and *WNT10B* are currently recognized. As a transcription factor, TP63 plays an important role in the development of the ectoderm.

**Methods:**

We identified a pathogenic variant by extracting DNA from the peripheral blood of a patient with SHFM and performing whole-exome sequencing (WES). After verifying the variant through Sanger sequencing, a series of analytical procedures were conducted to determine its pathogenicity. These included conservative analysis, tolerance analysis of the mutant region, and three-dimensional molecular modeling of the protein.

**Results:**

A *TP63* frameshift mutation (NM_003722; c.2009_2010insA; p.N670Kfs^*^) associated with SHFM was identified by WES and is predicted to cause a segmental deletion within the transactivation inhibitory domain (TID) of TP63. Bioinformatic analyses revealed that this locus is highly evolutionarily conserved and poorly tolerant of variation.

**Conclusion:**

We identified a *TP63* shift mutation in a patient with SHFM. This research contributes to the expansion of the spectrum of *TP63* variants and disease phenotypes, and is expected to provide valuable information for genetic counseling and prenatal diagnosis in families affected by similar congenital anomalies.

## 1 Introduction

Split-hand/foot malformation (SHFM) is a congenital limb deformity that predominantly affects the central rays of the hands and/or feet. The incidence of this disease ranges from 1/25000 to 1/8500, accounting for 8% to 17% of all limb deformities (1). SHFM exhibits a high degree of clinical heterogeneity. SHFM is associated with various congenital malformations, including ectodermal dysplasia-ectodermal dysplasia syndrome (EEC) (2) and brachydactyly-ectrodactyly with fibular aplasia or hypoplasia (3). Embryonic development is precisely regulated by interactions among multiple signaling

pathways, and the occurrence and progression of SHFM are influenced by multiple mechanisms. Consequently, the etiology of SHFM is multifactorial and involves the influence of numerous pathogenic genes. Currently, genes including *TP63, DLX5, DLX6, FGFR1, BHLHA9*, and *WNT10B* are known to be associated with SHFM (4–6). These genes exhibit relatively increased expression levels in the apical ectodermal ridge (AER), which is the primary signaling center responsible for regulating limb growth, and the associated pathways demonstrate notable conservation across different species (6).

*TP63*, a member of the p53 protein family, is characterized by high sequence and structural similarity to p53 and p73. As a transcription factor, TP63 regulates numerous physiological processes, including development, differentiation, and cell lineage specification (7). Diseases caused by TP63 defects includes EEC syndrome, Ankyloblepharon-Ectodermal Dysplasia-Clefting (AEC) syndrome, SHFM, Rapp-Hodgkin syndrome, orofacial cleft, limb-mammary syndrome, and ADULT syndrome (8–10). Patients with these diseases have overlapping characteristic clinical manifestations, mainly affecting organs and tissues derived from the ectoderm (11), such as abnormal hair (less hair or partial or complete hair loss), teeth (enamel defect or loss), nails (malnutrition, hypertrophy, or abnormal keratinization) or sweat glands (dysplasia or regenerative disorder) (12). However, these syndromes are frequently accompanied by some nonclassic features, which complicates direct diagnosis. These diseases are frequently characterized by severe and irreversible congenital dysplasia, which complicates their management. Genetic screening for *TP63* variants has been shown to have early diagnostic value for and significant clinical significance in the reduction of such congenital anomalies.

In this study, we collected data from an SHFM patient with tooth dysplasia and ectodermal dysplasia and identified a *TP63* variant (NM_003722: c.2009_2010insA, p.N670Kfs^*^). These findings expand the spectrum of *TP63* variants and associated phenotypes, providing a basis for further investigations and for the development of enhanced prenatal diagnostic approaches and genetic counseling protocols.

## 2 Materials and methods

### 2.1 Patients and subjects

This research was approved by the Review Board of Xiangya Hospital of Central South University (202103427). A 53-year-old male patient with SHFM and his family members were recruited after providing informed consent.

### 2.2 Whole exome sequencing and Sanger sequencing

Genomic DNA was extracted from peripheral blood samples from all the subjects using a DNeasy Blood & Tissue Kit (Qiagen, Valencia, CA, USA). The majority of whole-exome sequencing was performed by Berry Genomics Company Limited (Beijing, China). Data filtering strategies and the necessary bioinformatics analyses were performed as previously described (13, 14). Exome capture and high-throughput sequencing were performed by Berry Genomics Company. One milligram of DNA was randomly sheared using a Covaris S220 sonicator (Covaris, Inc., Woburn, USA), subjected to exon capture to generate a DNA library, and subjected to high-throughput sequencing using an Illumina HiSeq 2500 platform (Illumina, San Diego, CA, USA). The GnomAD (https://gnomad.broadinstitute.org/) and 1000G (https://www.internationalgenome.org) databases were used to remove variants with a population detection rate > 0.001 (a rare disease is defined by the WHO as a disease that affects 0.65–1% of the total population). The pathogenicity of the remaining variants was subsequently predicted by MutationTaster (https://www.mutationtaster.org/), PolyPhen-2 (http://www.org/index.php), SIFT (http://provean.jcvi.org/index.php), and CADD (https://cadd.gs.washington.edu/snv). We annotated candidate variants for inheritance patterns and clinical phenotypes through OMIM (https://www.omim.org) and classified them at the pathogenic level in accordance with the standards and guidelines of the American College of Medical Genetics and Genomics (ACMG).

Sanger sequencing was then used to verify the *TP63* (transcript: NM_003722) variant. The primer pair (TP63 5 → 3 f: GAGCGTGTTATTGATGCTGTG; TP63 5 → 3 r: ATTAGATGCCAGGTCAGATGTT) was designed by Integrated DNA Technologies (https://sg.idtdna.com/Primerquest/Home/Index) and used for polymerase chain reaction (PCR) to amplify the *TP63* target fragment. The DNA polymerase used in this study was the 2 × PCR MIX from TransGen Biotech. The thermocycling program for PCR was set as follows: initial denaturation at 95 °C for 3 min; denaturation at 95 °C for 30 s, annealing at 56.2 °C for 30 s, and extension at 72 °C for 15 s, for 30 cycles; and a final extension at 72 °C for 5 min. A 1% agarose gel was used, with GelRed as the visualization method.

### 2.3 Functional analysis of the mutant TP63 protein

The amino acid sequences were downloaded from NCBI (https://www.ncbi.nlm.nih.gov/protein/?term=TP63) for conservative analysis. The MetaDome web server (https://stuart.radboudumc.nl/metadome/dashboard) was used to assess the intolerance of TP63 to functional genetic variations. The intrinsically disordered protein regions (IDRs) in TP63 were predicted by PONDR (https://www.pondr.com/). The three-dimensional structure of TP63 (Q9H3D4) was obtained from the UniProt database (https://www.uniprot.org/). SWISS-MODEL (https://swissmodel.expasy.org/) was used to construct the p.N670Kfs^*^ mutant model based on the wild-type TP63 structure.

## 3 Results

### 3.1 Clinical phenotype of patient

**Figure 1:**
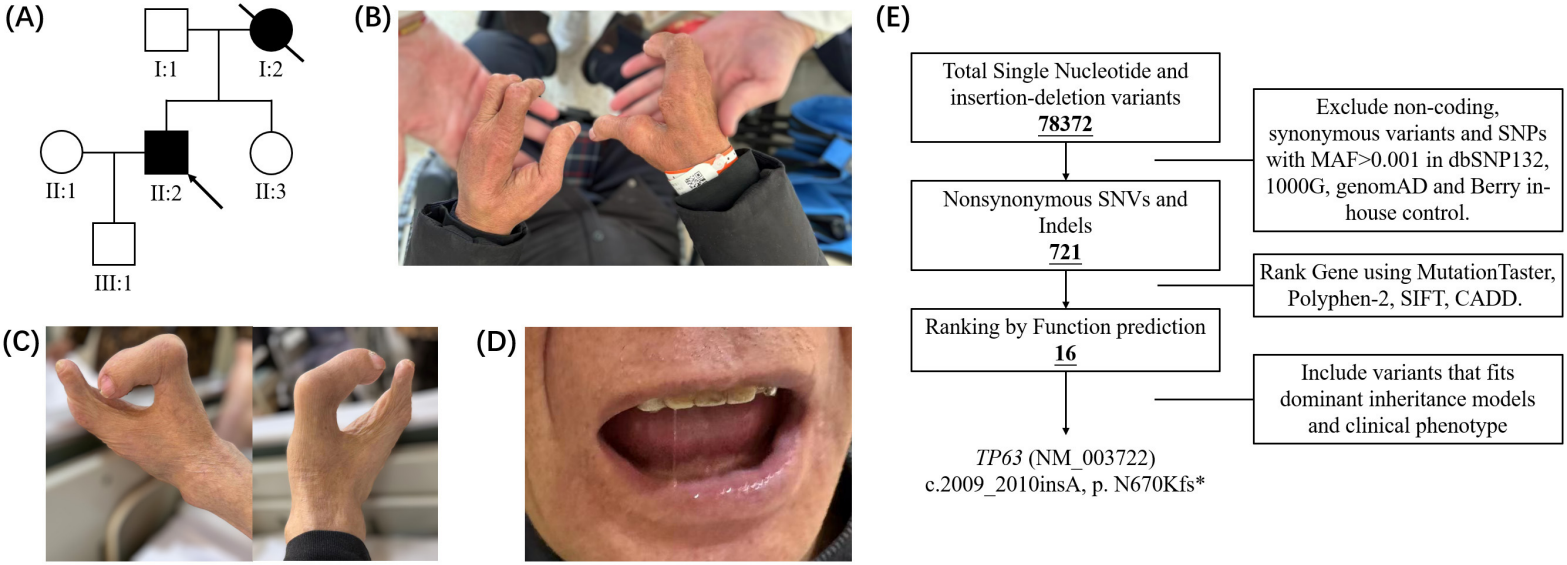
Lineage and phenotypes of proband. **(A)** Family members of proband and their medical conditions. **(B–D)** Clinical phenotype of proband. The findings show that the patient has SHFM and loss of lower teeth. **(E)** Filtering identified 1 candidate variant.

The proband (II:2; Figure 1A), a 53-year-old man, was found to have a congenital cleft hand and foot deformity characterized by the complete absence of the 2nd and 3rd fingers and metacarpal bones in both hands, a radially curved thumb, and complete syndactyly of the 4th and 5th fingers of the right hand (Figure 1B). The 2nd, 3rd, and 4th toes and metacarpal bone of both feet are absent, and the big toe is markedly flexed toward the midline of the foot (Figure 1C). Moreover, congenital dental dysplasia manifested as agenesis of the lower teeth, were observed (Figure 1D). Since the patient's reason for seeking medical attention was intellectual disability, specialized examinations of the hair, nails, skin, sweat glands, etc., were not conducted. No additional abnormalities were detected on comprehensive examination. A review of his family history indicated that in addition to his deceased mother (I:2), who had exhibited a similar phenotype, his father (I:1), sister (II:3), and son (III:1) all displayed normal phenotypes (Figure 1A). Accordingly, the proband was initially diagnosed with SHFM accompanied by congenital ectodermal dysplasia. Given the patient's advanced age, the suboptimal outcomes of previous corrective surgeries, and the elevated risk, surgical intervention was not recommended.

### 3.2 Genetic analysis

The proband (II:2) underwent whole-exome sequencing, with the target regions achieving more than 10 × coverage. Following comparison with single-nucleotide polymorphism (SNP) testing, a total of 78,372 variant sites were identified within the proband's genome. Following data filtering and assessment in accordance with the guidelines of the American College of Medical Genetics and Genomics (15) (Figure 1E), only a novel *TP63* variant (c.2009_2010insA, p.N670Kfs^*^) was classified as “Pathogenic” (Table 1), and no other variants met the criteria for “Pathogenic” or “Likely Pathogenic.” To explore the etiology of SHFM in the proband by known genetic mutation, we focused on the 37 remaining variants of the proband and revised our strategy using a filter of 16 SHFM and digital deformity-related genes (Supplementary Table S1). Single-patient analysis excluded the possibility that a known causative gene underlies SHFM.

**Table 1:** The *TP63* Variants identified in the proband by WES.

Gene	Variant	Pathogenicity prediction	GnomAD	1000G	OMIM clinical phenotype	ACMG classification
*TP63*	NM_003722, c.2009_2010insA, p.N670Kfs^*^	MutationTaster: D^i^ Polyphen2: - SIFT: - CADD: 34.2	-	-	AD, ADULT syndrome; AD, Ectrodactyly, ectodermal dysplasia, and cleft lip/palate syndrome 3; AD, Hay-Wells syndrome; AD, Limb-mammary syndrome; ?, Orofacial cleft 8; AD, Premature ovarian failure 21; AD, Rapp-Hodgkin syndrome; AD, Split-hand/foot malformation 4.	**Pathogenic: PVS1** (truncating mutation) + **PM1** (The variant is located in the TID domain of TP63) + **PM2** (The variant Variants not detected in the normal control populations of GnomAD and 1000G) + **PP1** (The variant co-segregates with the disease in the family)

D^i^, disease causing. ^*^Indicates a premature stop codon resulting from the frameshift mutation, leading to truncated protein production.

**Figure 2:**
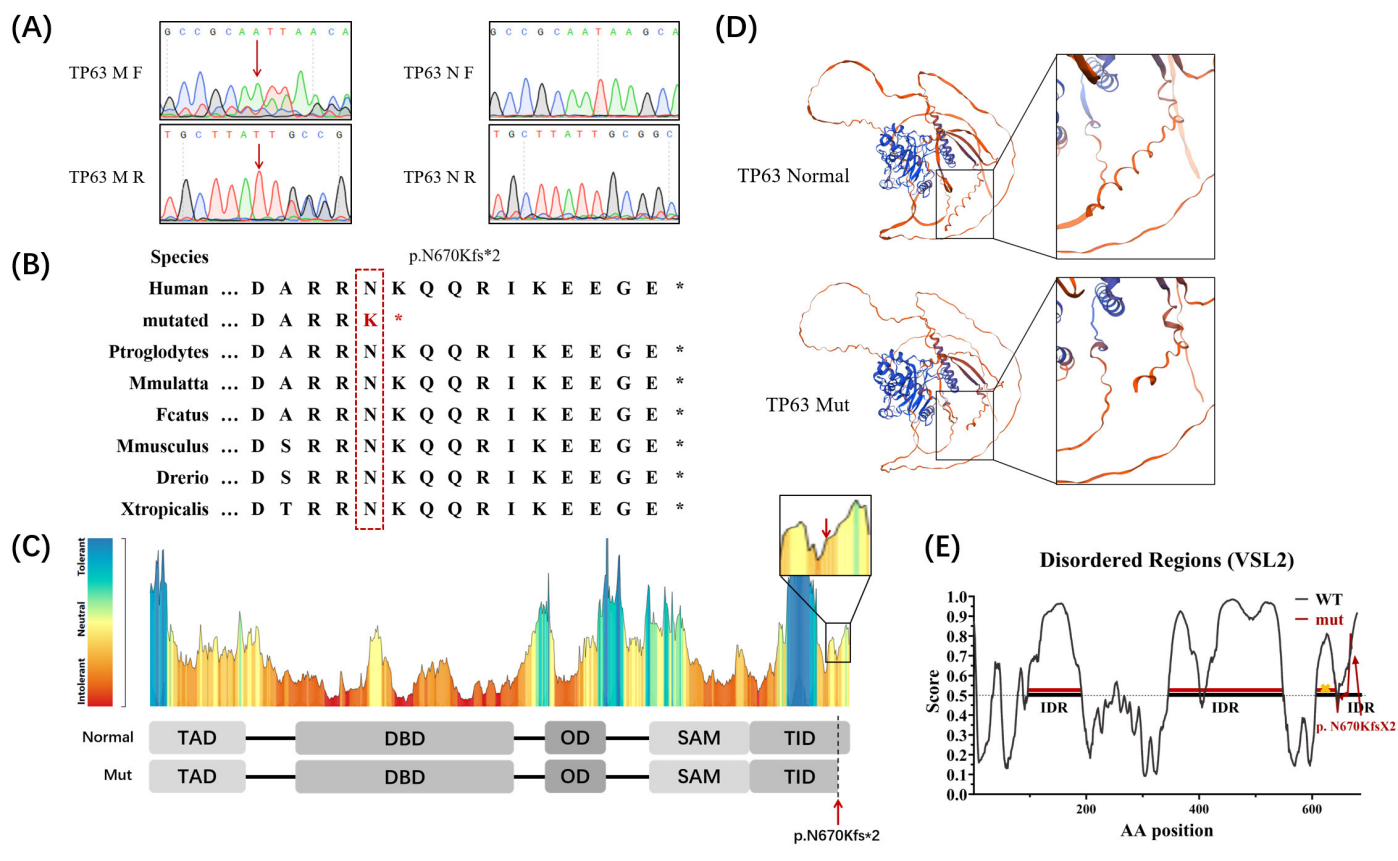
Sanger sequencing and bioinformatics analysis of TP63 mutation. **(A)** Sanger sequencing of patient and normal person. **(B)** Evolutionary conservation analysis of the TP63 mutation site. **(C)** Tolerance landscape of TP63 protein. **(D)** Structural of the wild-type and mutant TP63 proteins. **(E)** Deletion of an IDR segment of the TP63 mutant protein.

The variant is a frame-shift variant that causes the 670th amino acid (asparagine) to mutate into lysine, whereby the codon encoding the 671st amino acid (lysine) becomes a stop codon, and the subsequent amino acids are deleted, which conforms to PVS1. The variant is located in the TID domain of TP63, conforming to PM1. It is absent from the normal control populations of gnomAD and 1000G, conforming to PM2. And it co-segregates with the disease in the family, conforming to PP1. In conclusion, in accordance with the guidelines of ACMG, *TP63* (c.2009_2010insA, p.N670Kfs^*^) is classified as “Pathogenic” and can be attributed to the genetic etiology of the proband. The variant in the proband was verified by Sanger sequencing (Figure 2A), and other unaffected subjects did not harbor it.

Furthermore, cross-species alignment analysis of the amino acid sequences revealed that this mutation site and its truncated downstream region were highly conserved during evolution (Figure 2B) and that these loci in the TID domain were relatively intolerant to alterations in amino acids (Figure 2C). Three-dimensional structural models of the wild-type and mutant TP63 proteins constructed by SWISS-MODEL revealed that the C-terminal segment of the mutant protein was lost (Figure 2D). The deleted region was predicted to reside within an intrinsically disordered region (IDR), and its removal is expected to disrupt this IDR (Figure 2E).

## 4 Discussion

SHFM can present as an isolated malformation or can be associated with other congenital anomalies. The typical phenotype of SHFM includes the loss of one or more central fingers. The first and fifth fingers are usually preserved but malformed, and the remaining fingers are frequently fused (16). The developing limb bud is composed of two layers: a highly proliferative mesenchymal core and an ectodermal covering. The pattern of limb buds is a process that is mediated by signaling molecules produced by three specific cell groups: the apical ectodermal ridge (AER), the progressive zone (PZ), and the polarized active zone (ZPA) (17). Failure to maintain the AER has been demonstrated to affect the formation of the automatic foot and lead to the development of the SHFM phenotype (18). *TP63* plays a key role in the formation of the AER and the control of AER function through transcriptional regulation of AER-restricted target genes (including *Dlx5, Fgf8, Sp6, Sp8*, and *Msx1*). *TP63* has been shown to regulate the epithelial stratification process and to control the formation and differentiation of the AER by regulating AER restriction genes (19). It has been demonstrated that mice lacking the *Tp63* gene are incapable of forming normal ectodermal structures; this is characterized by the abnormal development of skin, hair, teeth, craniofacial bones, breasts, and limbs (20–22). In our study, the ectodermal dysplasia observed in the proband was confined to the limbs and teeth.

TP63 has been identified as a transcription factor that is homologous to p53 and p73 (23). The biological functions of TP63 are mediated by multiple functionally distinct isoforms. Two major transcripts—TAp63 and ΔNp63—are generated from *TP63* via alternative promoter usage. TAp63 contains a transcriptionally activating domain (TAD), a DNA-binding domain (DBD), an oligomerization domain (OD), a sterile alpha motif (SAM), and a TID, and ΔNp63 lacks an N-terminal TAD. Each can give rise to three isoforms, α, β, and γ, because of the differences in carboxyl-terminal splicing (24). All of these isoforms have a DBD and an OD, but different isoforms possess various regions on the C-terminal (25, 26). Our *TP63* variant (c.2009_2010insA, p.N670Kfs^*^) destroyed the integrity of certain TP63 isoforms. Premature termination of the mutant protein affected the TID and led to the absence of the last IDR (Figure 2E). Studies have shown that in epithelial tissues, TID synergizes with SAM to direct ΔNp63α to preferentially bind distal enhancers of target genes. Here, ΔNp63α mainly interacts with chromatin remodeling complexes including SMARCC2, KMT2D, and HDAC1/2, moderately activating proliferation-associated genes by regulating chromatin 3D structure and accessibility. Additionally, TID's transcriptional repressive activity restricts ΔNp63α from interacting with general transcription factors such as GTF2i, GTF2A1, and RNA polymerase II, thus preventing its excessive binding to and aberrant activation of proliferation-associated gene promoters. Moreover, TID-mediated transcriptional repression abrogates sustained robust activation of differentiation repressor genes by ΔNp63α, enabling cells to respond to exogenous differentiation signals like Ca^2+^ signals and sequentially express the differentiation markers K10, loricrin, and involucrin (27). In the absence of TID, ΔNp63α instead binds proximal promoters of target genes, causing excessive transcriptional activation of proliferation-associated genes and concomitant inhibition of normal differentiation signal initiation (28). We speculate that this mutation may impair the function of the TID, which is a key regulatory domain for ΔNp63α-mediated epithelial cell proliferation and differentiation balance; however, further functional assays are needed to validate this hypothesis and elucidate the precise underlying molecular mechanism.

Variants located on different structural domains often exhibit different phenotypic characteristics. For example, ectrodactyly–ectodermal dysplasia–cleft lip and palate (EEC) syndrome, the prototype of the *Tp63* family of syndromes, is caused by missense variants occurring predominantly in the 1/5 arginine (R) “hotspot” located in the DNA-binding domain of Tp63 (29). Among them, 80% of patients with R304 variants have cleft lips and palates, while those with R227 variants rarely have facial clefts (23). Ankyloblepharon–ectodermal dysplasia–facial cleft syndrome (AEC) and Rapp–Hodgkin syndrome (RHS) can be considered a single syndrome, and their phenotypic manifestations differ in severity. The typical variants observed in AEC/RHS cases occur in the SAM or the TID (30). The common variant in patients with ADULT syndrome is located in the middle of the DNA binding domain, and studies have shown that this may affect the binding of TP63 to DNA (31). The TP63 variant in this study was located in the TID. The proband exhibited symptoms consistent with ectodermal dysplasia in addition to SHFM. However, the reason why patients do not exhibit the classic RHS phenotype is unknown. Although no typical ectodermal dysplasia phenotypes were identified during routine clinical assessment, specialized and systematic examinations of the hair, nails, skin, and sweat glands were not performed. Accordingly, the possibility of mild or occult ectodermal involvement cannot be definitively excluded, representing a potential limitation of this study. Several variants have also been reported in the vicinity of the loci we identified, including nonsense variants at the 658th, 673rd, and 678th amino acids, which also contribute to the SHFM phenotype (16, 23, 32).

In these TP63-related disorders, certain syndromes have strong genotype–phenotype correlations, whereas others have unclear correlations, possibly representing additional and complex potential genetic defects and modified alleles. Consequently, the collection of additional cases of *TP63* variants and their corresponding phenotypes is instrumental for elucidating the molecular mechanisms underlying this particular congenital malformation from multiple perspectives. This study may contribute to improved genetic counseling and precise prenatal screening for SHFM and related disorders.

## 5 Conclusions

Through genetic analysis, a novel *TP63* variant (NM_003722, c.2009_2010insA, p.N670Kfs^*^) was identified in a patient with SHFM with tooth loss. Our findings expand the genetic and phenotypic spectrum of *TP63* variants, providing some insight for subsequent genetic counseling and new perspectives for clinical diagnosis.

## Data Availability

The datasets presented in this study can be found in online repositories. The names of the repository/repositories and accession number(s) can be found in the article/Supplementary material.
