# Supplementary material for: Identification of novel tumor protein 63 variant associated with split-hand/foot malformation and tooth agenesis
Source: Front Med (Lausanne). 2026 Apr 24;13:1811133. doi: 10.3389/fmed.2026.1811133 (PMC13152756; doi:10.3389/fmed.2026.1811133)
Supplement: Supplementary file 1 [file Table_1.docx]

**Table S1.** 16 SHFM and digital deformity-related genes for filter

| TP63 | DLX5 | DLX6 | FGFR1 |
| --- | --- | --- | --- |
| BHLHA9 | WNT10B | GLI1 | GLI3 |
| HOXA13 | HOXD13 | BMP4 | FGFR2 |
| BMPR1B | LMBR1 | GJA1 | LRP4 |
